# Lens capsule advanced glycation end products induce senescence in epithelial cells: Implications for secondary cataracts

**DOI:** 10.1111/acel.14249

**Published:** 2024-06-21

**Authors:** Grace Cooksley, Mi‐Hyun Nam, Rooban B. Nahomi, Johanna Rankenberg, Andrew J. O. Smith, Yvette M. Wormstone, I. Michael Wormstone, Ram H. Nagaraj

**Affiliations:** ^1^ Department of Ophthalmology, School of Medicine University of Colorado Aurora Colorado USA; ^2^ School of Biological Sciences University of East Anglia Norwich UK; ^3^ Nottingham Ningbo China Beacons of Excellence Research and Innovation Institute University of Nottingham Ningbo China Ningbo China; ^4^ Department of Pharmaceutical Sciences, Skaggs School of Pharmacy and Pharmaceutical Sciences University of Colorado Aurora Colorado USA

**Keywords:** advanced glycation end products, epithelial‐mesenchymal transition, lens epithelial cells, posterior capsule opacification, senescence

## Abstract

Posterior capsule opacification (PCO) is a common complication after cataract surgery. Residual lens epithelial cells (LECs) on the anterior lens capsule, after cataract surgery, migrate to the posterior lens capsule and undergo transdifferentiation into myofibroblast‐like cells. Those cells synthesize excessive amounts of extracellular matrix and contribute to fibrosis during PCO. Cellular senescence, a phenomenon that increases with aging, has been implicated in several fibrotic diseases. Here, we have investigated the prevalence of senescent LECs within the lens posterior capsule and the ability of advanced glycation end products (AGEs) in lens capsules to induce senescence, contributing to PCO. Aged lens capsules from pseudophakic human cadaver eyes showed the presence of senescent LECs. In human capsular bags, LECs showed an age‐dependent increase in senescence after 28 days of culture. Human LECs cultured on aged lens capsules for 3 days underwent senescence; this effect was not seen in LECs cultured on young lens capsules. Human LECs cultured on an AGE‐modified extracellular matrix (ECM‐AGEs) showed an AGE‐concentration‐dependent increase in the expression of senescence markers and reactive oxygen species (ROS) levels. Treatment with a RAGE antagonist and ROS inhibitor reduced the expression of senescence and fibrotic markers. Additionally, conditioned media from ECM‐AGEs‐treated cells induced the expression of fibrotic markers in naïve LECs. Together, these suggest that AGEs in the capsule induce senescence of LECs, which triggers the mesenchymal transition of neighboring non‐senescent LECs and contributes to PCO.

## Graphical abstract

Advanced glycation end products (AGEs) in the posterior capsule of the lens induce senescence of lens epithelial cells (LECs). The senescent LECs secrete senescence‐associated secretory phenotype (SASP) and promote epithelial‐to‐mesenchymal transition of non‐senescent LECs. These events could potentially contribute to fibrosis during the development of posterior capsule opacification after cataract surgery in elderly patients.

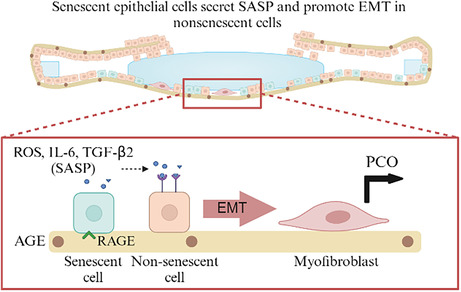

**Abbreviations**
α‐SMA: α‐smooth muscle actin
AGEs: advanced glycation end products
ECM: extracellular matrix
EMT: epithelial‐mesenchymal transition
IL‐6: interleukin‐6
IOL: intraocular lens
LECs: lens epithelial cells
MMP2: matrix metalloproteinase‐2
Nd:YAG: neodyium‐doped yttrium aluminium garnet
PCO: posterior capsule opacification
RAGE KO: RAGE knock‐out
RAGE: receptor for advanced glycation end products
ROS: reactive oxygen species
SASP: senescence‐associated secretory phenotype
SA‐β‐gal: senescence‐associated beta‐galactosidase
TGF‐β2: transforming growth factor beta
WT: wild‐type

## 1 INTRODUCTION

Cataracts are the primary cause of blindness, with approximately 15 million people worldwide blind in 2020 (Blindness, Vision Impairment, & Vision Loss Expert Group of the Global Burden of Disease, 2021; Lee & Afshari, 2017). Although surgical removal of cataracts and implantation of intraocular lens (IOL) restores vision in most patients, it can lead to complications such as endophthalmitis, cystoid macular edema, retinal detachment, and posterior capsule opacification (PCO) (Chan et al., 2010; Cooksley et al., 2021; Stein, 2012; Wormstone et al., 2021). PCO involves abnormal differentiation and transformation of lens epithelial cells (LECs), allowing them to migrate and colonize the previously acellular posterior capsule. During cataract surgery, most capsule adherent LECs are removed. However, despite best efforts, some LECs remain attached. The residual equatorial LECs can proliferate and form a fiber cell‐like structure that eventually forms a ring between the posterior capsule and the borders of the anterior capsule remnants, known as Soemmering's ring (D'Antin et al., 2022; Jongebloed et al., 1988; Wormstone et al., 2021). Some residual LECs can also become poorly differentiated and form grape‐like clusters known as Elschnig's pearls at the equator of the capsule (Findl et al., 2010; Raj et al., 2007; Wormstone et al., 2021). These cell transformations contribute to a regenerative form of PCO.

In fibrotic PCO, cells that migrate to the posterior capsule undergo an epithelial‐to‐mesenchymal transition (EMT). Many studies have shown that transforming growth factor‐β2 (TGF‐β2) is the major driver of EMT of LECs (reviewed in Wormstone et al., 2021). The cells that have undergone EMT upregulate mesenchymal markers such as α‐smooth muscle actin (α‐SMA) and fibronectin and secrete extracellular matrix (ECM) proteins that contribute to fibrosis associated with PCO. In addition, TGF‐β is reported to promote matrix contraction (Wormstone et al., 2002).

PCO develops in many patients after cataract surgery. As a result, patients' vision is compromised. Nd:YAG laser capsulotomy is performed to remove the fibrous material to clear the visual pathway. The incidence of Nd:YAG laser capsulotomy ranges between 2.4% to 12.6% at 3 years and 5.8% to 19.3% at 5 years after cataract surgery (Ursell et al., 2020). However, this technique occasionally leads to complications, such as retinal tear/detachment, cystoid macular edema, and a rise in intraocular pressure (Karahan et al., 2014). There are no therapies to prevent or reverse PCO. Further research on PCO is required to expand our understanding of the underlying molecular mechanisms and prevent its formation.

The lens capsule is a basement membrane secreted by LECs; the proteins in the capsule do not turnover and, therefore, accumulate many age‐related modifications. Glycation is a post‐translational modification involving the reaction of reactive aldehydes and carbonyl compounds with amino groups of proteins. This reaction produces various chemical modifications in proteins collectively known as advanced glycation end products (AGEs) (Ruiz et al., 2020). We have previously shown that AGEs accumulate in aging human lens capsules and at a higher rate in cataractous lenses (Raghavan et al., 2016). We have also shown that AGEs in lens capsules enhance the TGF‐β2‐mediated EMT of LECs (Raghavan & Nagaraj, 2016). Based on these findings, we proposed that capsule AGEs bind to their receptor (RAGE) in LECs, initiating pathways leading to PCO.

The senescence of LECs has been implicated in cataract formation (Wang et al., 2022; Yan et al., 2019), although mechanisms are unclear. Glycation is a factor for cellular senescence in several cell types, and such senescence has been implicated in age and diabetes‐associated complications (Zheng et al., 2022). Upon telomere loss or DNA damage, cells exit the cell cycle at G0 and undergo senescence. This is accompanied by the upregulation of cyclin‐dependent kinases p21 and p16 (Lee et al., 2006). Senescent cells produce ROS, senescence‐associated beta‐galactosidase (SA‐β‐gal) (Lee et al., 2006), and secrete senescence‐associated secretory phenotype (SASP) (Cuollo et al., 2020). SASP consists of many factors, including growth factors and cytokines (Cuollo et al., 2020). Ineffective clearance of senescent cells results in the influx of SASP contents to local tissue, contributing to diseases (Cuollo et al., 2020). Whether senescence and SASP play a role in PCO formation and whether AGEs in lens capsules induce LEC senescence have yet to be investigated. This study has shown that aged pseudophakic eyes contain senescent LECs on the posterior lens capsules and that ECM‐AGEs promote the senescence of human LECs in vitro. The study also demonstrated that ECM‐AGEs‐induced senescence facilitates the EMT response in neighboring non‐senescent LECs, suggesting a potential mechanism for PCO. This process is inhibited by the treatment with a RAGE antagonist, suggesting a potential mechanism to prevent PCO.

## 2 RESULTS

### 2.1 Senescent cells are present in the posterior lens capsule of human pseudophakic eyes

**FIGURE 1:**
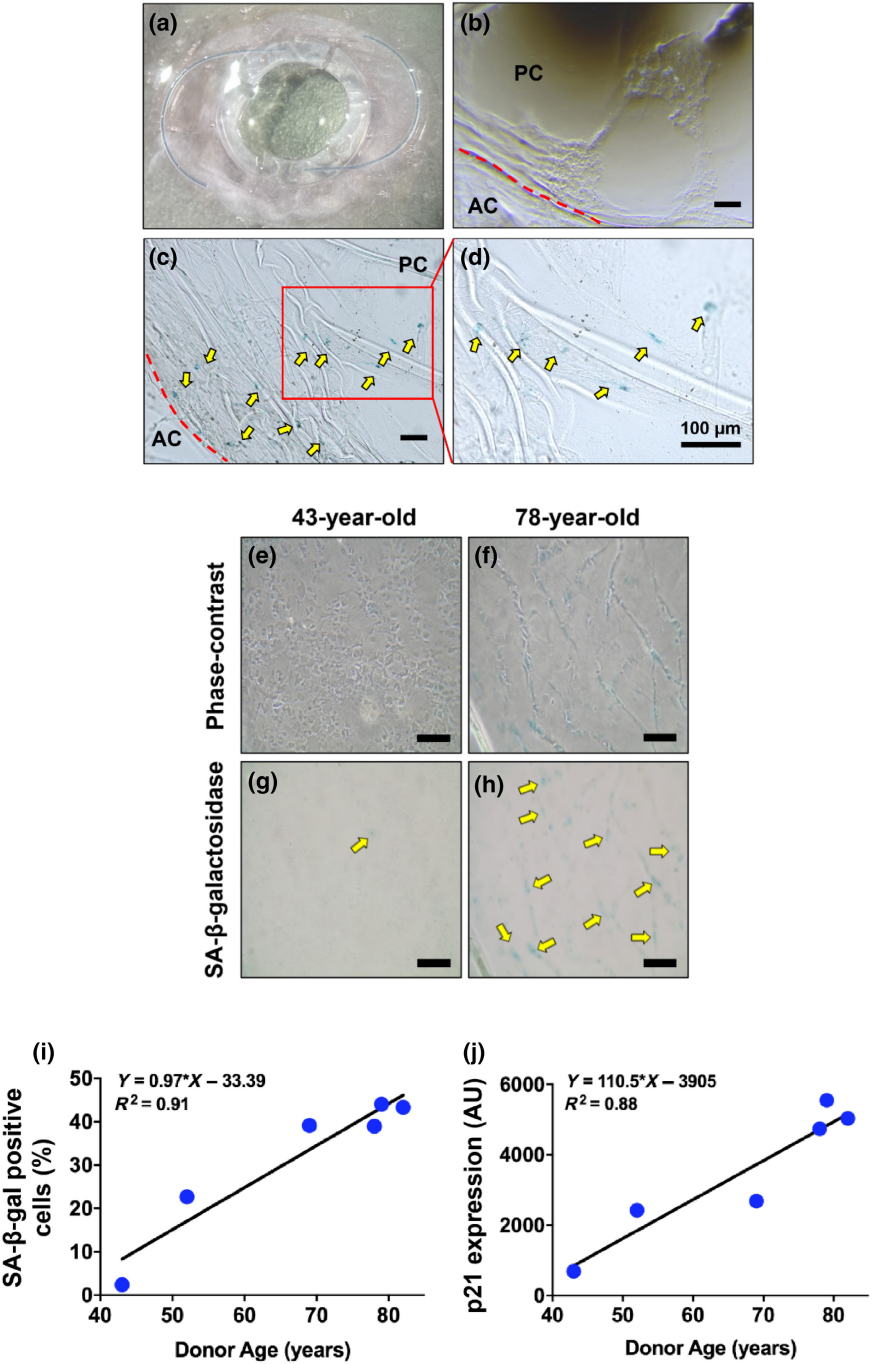
Senescent cells are present in the posterior lens capsule of pseudophakic eyes from patients and in vitro human capsular bag cultures. Representative image of an IOL‐containing capsule isolated from a 73‐year‐old pseudophakic human eye (a). Senescent cells were detected by senescence‐associated beta‐galactosidase (SA‐β‐gal)‐staining. A representative image of a posterior capsule lacking senescent cells in a pseudophakic eye (65‐year‐old) (b). A representative image showing senescent cells (indicated by arrows) in the posterior capsule of a 73‐year‐old (c). The enlarged image of C shows senescent cells at matrix contraction sites (d). Representative images show phase‐contrast and SA‐β‐gal positive senescent cells cultured under serum‐free conditions in 43‐ and 78‐year‐old capsular bags for 28 days (e–h). LECs cultured in six capsular bags from 43‐ to 82‐year‐old donors were quantified for the percentage of SA‐β‐gal‐stained cells (i) and p21 levels (j). Scale bars = 100 μm.

A lens capsule containing an IOL, isolated from a 73‐year‐old pseudophakic donor eye, is shown in Figure 1a. After IOL removal of the 12 pseudophakic eyes, seven showed cells in the posterior capsule, and a representative image (65‐year‐old) is shown in Figure 1b. The lens capsules were stained for SA‐β‐gal to detect senescent cells. Among the seven capsules containing LECs, four capsules showed SA‐β‐gal positive cells. A representative capsule (73‐year‐old) with senescent cells in the posterior capsule is shown in Figure 1c. The enlarged image of Figure 1c showed senescent cells within the matrix contraction sites of the capsules (Figure 1d). These data suggest that LECs that migrate to the posterior capsule in pseudophakic eyes can undergo senescence and induce matrix contraction.

### 2.2 LEC senescence is capsule age‐dependent

Next, we assessed whether LECs in human lens capsular bags undergo senescence and whether that is capsule age‐dependent. During the 28 days of culture, cells gradually covered the central posterior capsule. Representative images of a 43‐ and 78‐year‐old capsule are shown; cells in the 78‐year‐old capsule exhibited a senescent phenotype, as indicated by positive SA‐β‐gal staining and at the sites of matrix contraction (Figure 1e–h). In contrast, the number of SA‐β‐gal positive cells was lower in the central posterior capsule of the 43‐year‐old donor lens capsular bag (Figure 1e,g). Further, we observed a direct correlation between SA‐β‐gal positive cells and the age of the capsular bags (Figure 1i). In addition, the expression of p21 in LECs positively correlated with the age of the lens capsular bags (Figure 1j). These findings suggest that the senescence of LECs is promoted in aged lens capsules and that the number of senescent cells is directly correlated with the age of the lens capsule.

### 2.3 Aged lens capsules promote senescence of LECs

**FIGURE 2:**
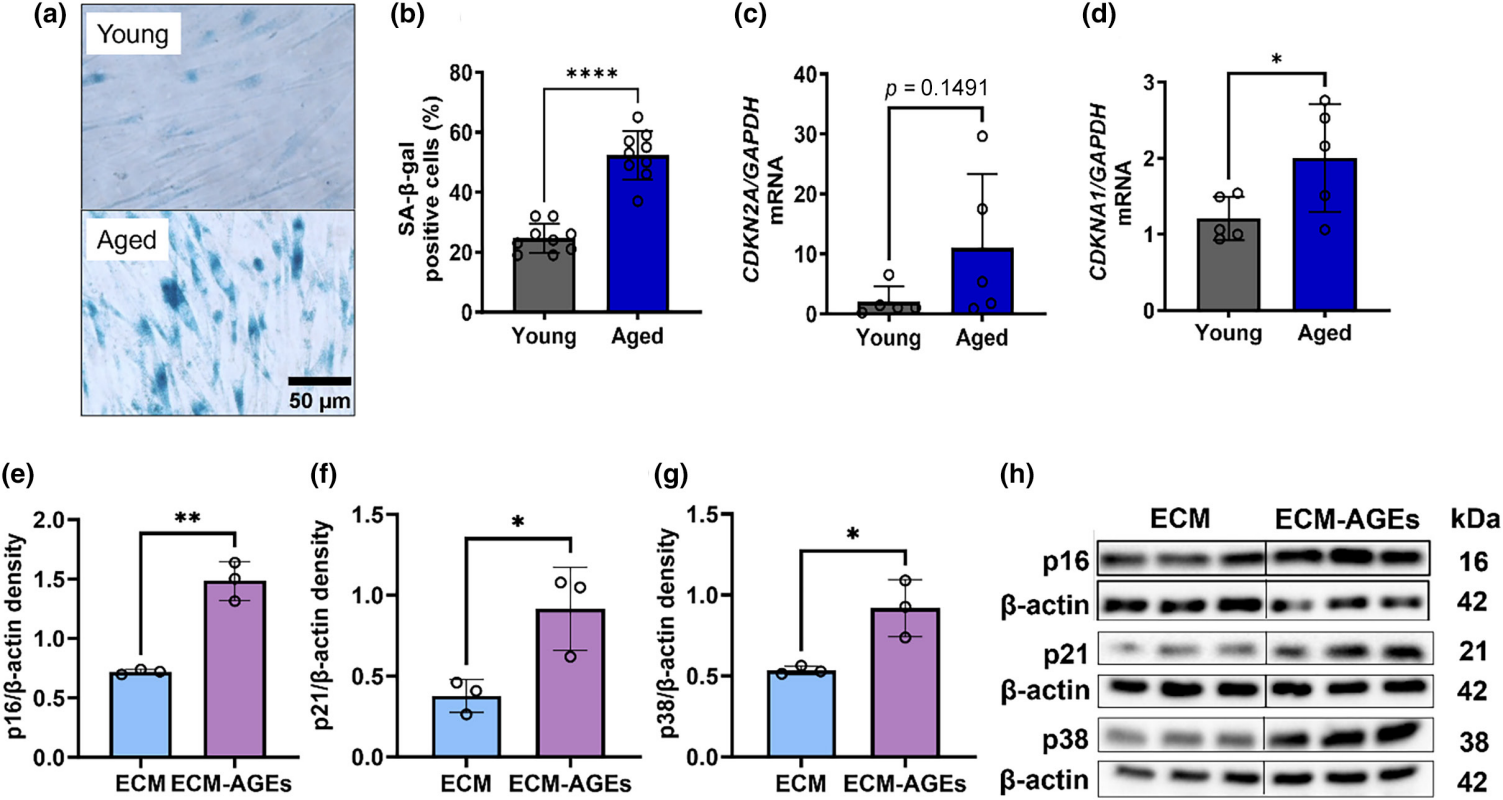
Aged human lens capsules and ECM‐AGEs promote the senescence of LECs. Human primary LECs (from a 16‐year‐old lens) were seeded on acellular young (19‐year‐old) and aged (72‐year‐old) posterior capsules, and after 3 days, cells were screened for senescence markers. Representative microscopic images show SA‐β‐gal positive cells (blue) (a), and the bar graph shows the percentage of SA‐β‐gal positive cells (b). Cells were cultured on young (17‐ to 22‐year‐old, *n* = 9) and aged (71‐ to 73‐year‐old, *n* = 9) capsules for which *CDKN2A* (p16) and *CDKN1A* (p21) mRNA levels were measured at 48 h and normalized to *GAPDH* (c, d, *n* = 5). FHL124 cells were cultured on ECM or ECM‐AGEs for 96 h. The expressions of p16 (e), p21 (f), and p38 (g) in the cell lysates were measured by western blotting (h) and normalized to the β‐actin loading control (*n* = 3). Data are mean ± SD of the indicated number of independent experiments. *p < 0.05; **p < 0.01; ****p < 0.0001. Scale bar = 50 μm.

The observations above suggest that aged lens capsules promote LEC senescence. To further investigate this, we cultured primary LECs on either aged (71‐ to 73‐year‐old) or young (17‐ to 22‐year‐old) lens capsules. After the removal of any adherent epithelial cells, primary human LECs isolated from a young 16‐year‐old lens were seeded onto the cell‐free capsules and cultured for 3 days. The SA‐β‐gal staining showed significantly higher staining of cells cultured on aged capsules than on young capsules (*p* < 0.0001) (Figure 2a,b). The qPCR results showed considerably higher mRNA levels for *CDKN2A* (p16) (*p* = 0.149) and *CDKN1A* (p21) (*p* < 0.05) in cells cultured on aged capsules than on young capsules (Figure 2c,d). These results further support the idea that aged capsules promote senescence of LECs.

### 2.4 ECM‐AGEs promote LEC senescence

We determined whether AGEs in lens capsules promote LEC senescence using AGE‐modified ECM. Our previous work (Raghavan et al., 2016) showed that the AGE content of modified ECM is comparable to that of aged human lens capsules. ECM was glycated using a glycating mixture for 7 days to generate AGEs. We found that glycated ECM did not significantly reduce cell viability or impair the binding of FHL124 cells (Figure S1). FHL124 cells cultured on ECM‐AGEs for 96 h showed significantly higher expressions of senescent markers p16 (*p* < 0.01), p21 (*p* < 0.05), and p38 (*p* < 0.05) relative to cells cultured on unmodified ECM (Figure 2e–h). The results suggest that the senescence of LECs observed in aged lens capsules is likely due to the presence of AGEs.

### 2.5 ROS formation and senescence in LECs are dependent on AGE levels

**FIGURE 3:**
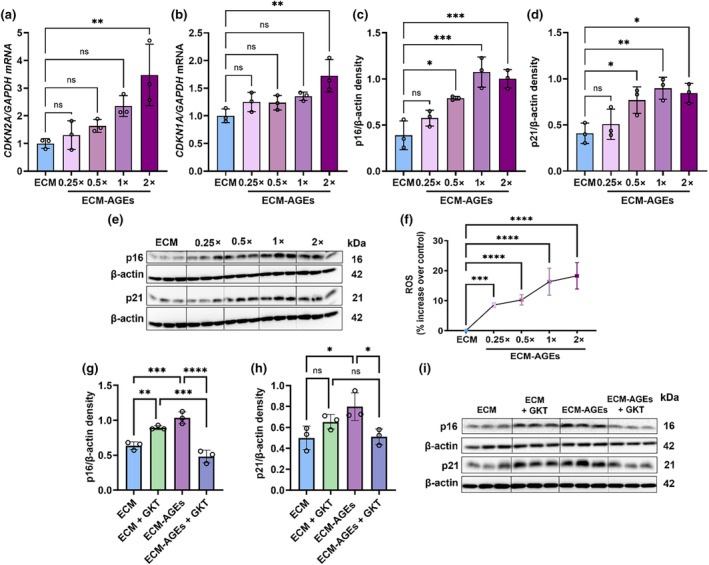
LEC senescence is AGE‐level and ROS‐dependent. ECM was coated in tissue culture wells for 24 h and incubated with or without a glycation mixture (0.25, 0.5, 1, 2×) for 7 days. FHL124 cells were cultured on ECM‐AGEs for 48 h to assess the mRNA levels of *CDKN2A* (p16) and *CDKN1A* (p21) (a, b), for 96 h to assess the expression of p21 and p16 protein (c–e) and for 24 h to measure ROS production (f). FHL124 cells on ECM or ECM‐AGEs were treated with or without 250 μM GKT136901 (GKT) for 72 h, and p16 and p21 expressions were measured by western blotting (g–i). Data are mean ± SD of three independent experiments. ns, not significant, *p < 0.05, **p < 0.01, ***p < 0.001, ****p < 0.0001.

We next determined the relationship between ECM‐AGE levels and LEC senescence. As AGE concentration increased, the mRNA levels (Figure 3a,b) and protein expression of *CDKN2A/*p16 and *CDKN1A*/p21 increased (Figure 3c–e). As ROS production has been linked to AGE‐RAGE interaction and the induction of senescence through NOX4 (Ott et al., 2014), we also investigated the impact of AGEs on ROS levels. With the increase in AGE concentration, there were significant elevations in ROS levels (Figure 3f). LECs cultured on 1X ECM‐AGEs showed 17% greater ROS generation over ECM control (*p* < 0.0001). To determine the role of ROS in AGE‐mediated LEC senescence, we examined the effects of a NOX4 inhibitor in FHL124 cells cultured on ECM‐AGEs. First, we determined the optimal concentration of the NOX4 inhibitor GKT136901 to inhibit AGE‐induced ROS formation. Our results showed that GKT136901 decreased AGE‐mediated ROS production dose‐dependently up to 150 μM, which showed a 33% reduction in ROS after 24 h (Figure S2). Higher concentrations of GKT136901 did not further reduce the ROS levels. At 150 μM concentration of GKT136901, we did not observe a significant reduction in ECM‐AGEs mediated senescent markers. However, when used at 250 μM concentration, a significant decrease in ECM‐AGEs mediated upregulation of p16 (*p* < 0.05) and p21 (*p* < 0.05) (Figure 3g–i) was observed, suggesting that ROS is a driver of AGE‐mediated senescence of LECs, but other mechanisms could contribute.

### 2.6 AGE‐induced senescent cells secrete TGF‐β2, IL‐6 and MMP‐2

**FIGURE 4:**
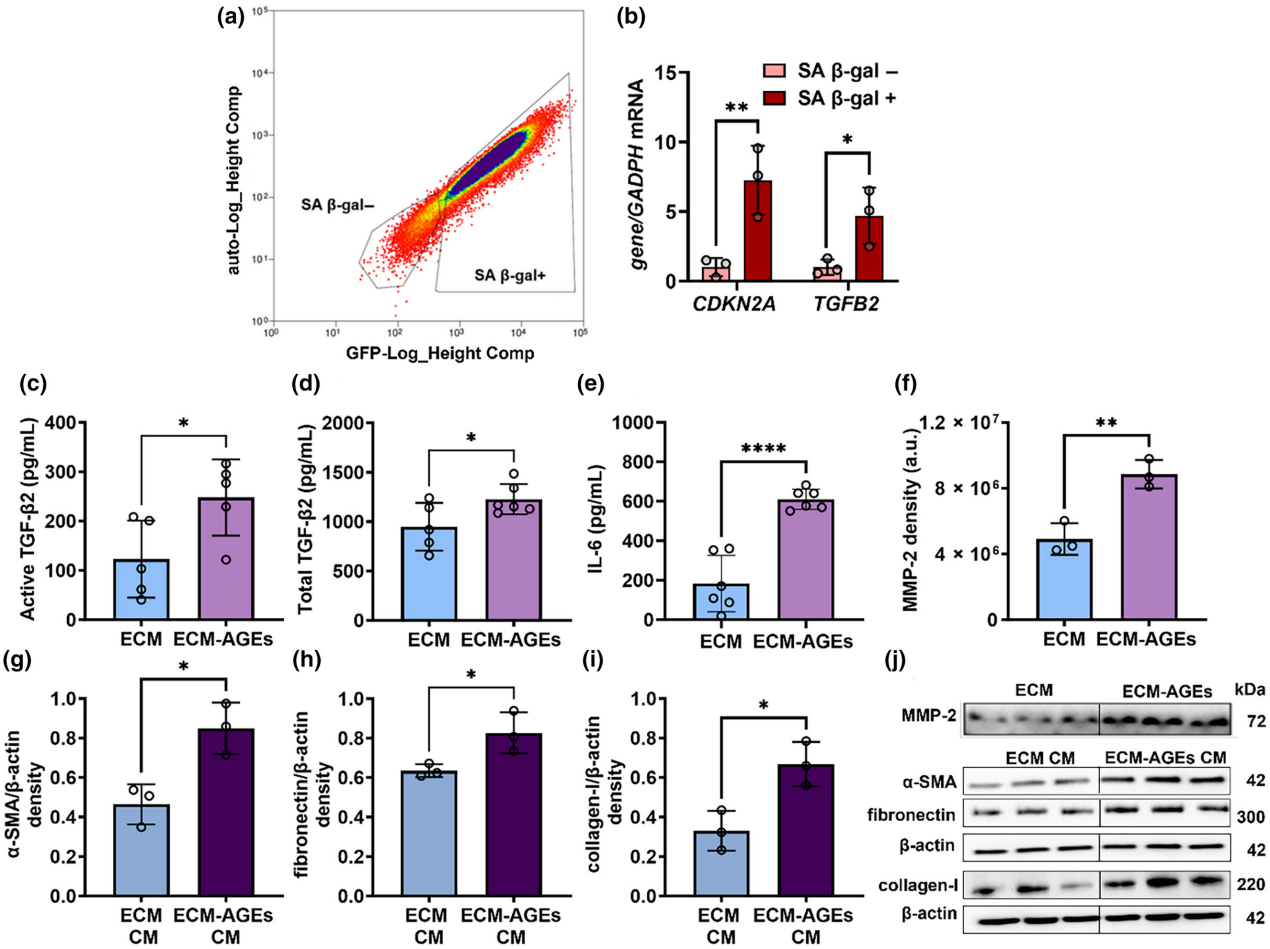
ECM‐AGEs‐induced senescent LECs secrete TGF‐β2 and IL‐6 and promote EMT in non‐senescent LECs. FHL124 cells were cultured on ECM‐AGEs for 96 h and sorted into SA‐β‐gal positive (+) and SA‐β‐gal negative (−) populations using flow cytometry (a). *CDKN2A* and *TGFB2* mRNA levels were measured in the sorted cells (b). Active and total TGF‐β2 (c, d, *n* = 5–6) and IL‐6 (e, *n* = 6) were measured in the conditioned media (CM) collected from cells cultured on ECM or ECM‐AGEs after 96 h by ELISAs. MMP‐2 was measured in CM collected from FHL124 cultured on ECM‐AGEs (f, *n* = 3). FHL124 cells were treated with CM from cells cultured on ECM (ECM CM) or ECM‐AGEs (ECM‐AGEs CM). After a 72 h incubation, the expression of α‐SMA (g), collagen I (h), and fibronectin (i) were measured in the cell lysate by western blotting (j, *n* = 3). Data are mean ± SD of the indicated number of independent experiments. *p < 0.05; **p < 0.01; ****p < 0.0001.

Cells cultured on ECM‐AGEs were separated into senescent and non‐senescent populations using SA‐β‐gal staining followed by flow cytometry (FACS) to determine whether the AGE‐induced senescence promotes EMT response. The FACS analysis revealed that the cell population cultured on ECM‐AGEs primarily comprised senescent cells (Figure 4a), supported by significantly higher levels of *CDKN2A* (p16) in the SA‐β‐gal positive cell population (*p* < 0.01) (Figure 4b). The *TGFΒ2* mRNA levels were also significantly higher in the senescent cell population when compared to the non‐senescent cell population (*p* < 0.05) (Figure 4b). This finding was further confirmed by comparing TGF‐β2 release from cells grown on ECM versus those grown on ECM‐AGEs. Higher levels of active (Figure 4c) and total TGF‐β2 (Figure 4d) were detected in the conditioned media of cells grown on ECM‐AGEs. We also measured the release of IL‐6, which has been shown to work in conjunction with TGF‐β2 to promote PCO (Ma et al., 2018). The results showed a significant (*p* < 0.0001) upregulation of IL‐6 release after 96 h of incubation when cells were cultured on ECM‐AGEs compared to those cultured on ECM (Figure 4e). Additionally, FHL124 cells cultured on ECM‐AGEs showed a significant increase in MMP‐2 release (*p* < 0.01) relative to those cultured on ECM (Figure 4f). We investigated whether SASP secreted by AGE‐induced senescent LECs promotes EMT in non‐senescent LECs in a paracrine manner. FHL124 cells were incubated with the conditioned media collected from cells cultured on ECM or ECM‐AGEs for 72 h. There was a significant upregulation in α‐SMA (*p* < 0.05), fibronectin (*p* < 0.05), and collagen I (*p* < 0.05) when cells were incubated with the conditioned media collected from cells grown on ECM‐AGEs compared to those incubated with the conditioned media from cells grown on unmodified ECM (Figure 4g–i). These results suggest that senescent LECs can induce EMT in non‐senescent LECs through a SASP‐mediated paracrine mechanism.

### 2.7 A RAGE inhibitor suppresses senescence and EMT markers in ECM‐AGE‐treated cells

**FIGURE 5:**
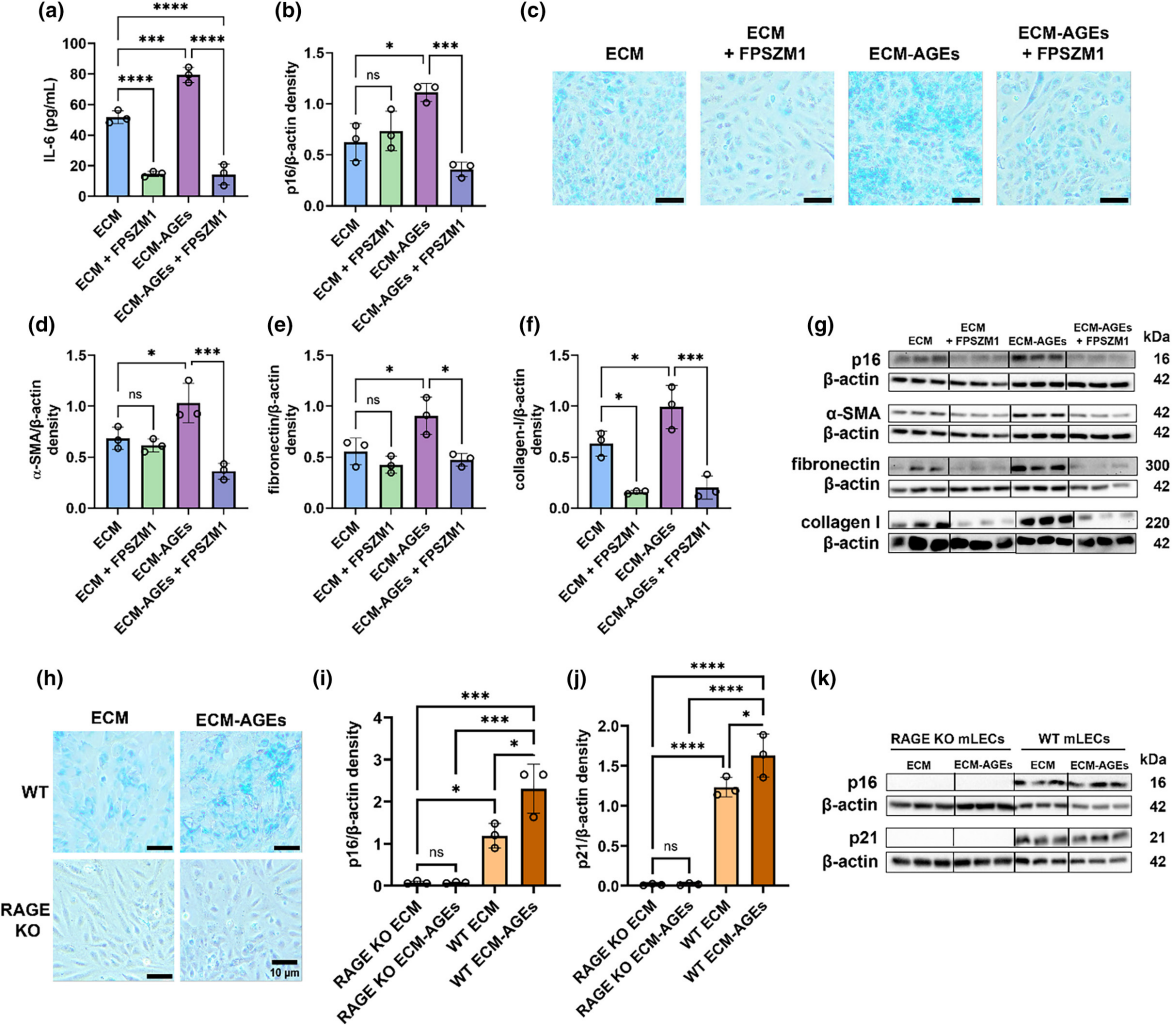
A RAGE antagonist inhibits senescence and EMT of LECs cultured on ECM‐AGEs. FHL124 cells were cultured on ECM or ECM‐AGEs. After 24 h, cells were treated with or without 20 μM RAGE antagonist FPSZM1 for an additional 72 h, then IL‐6 was measured from the collected media (a), and p16 was measured in cell lysates (b). Senescent cells were detected by senescence‐associated beta‐galactosidase (SA‐β‐gal)‐staining. Representative images of cells grown on ECM, ECM and FPSZM1 treatment, ECM‐AGEs, and ECM‐AGEs with FPSZM1 treatment (c) are shown. The expression of α‐SMA (d), fibronectin (e), and collagen I (f) were measured in cell lysates by western blotting (g). Wild‐type (WT) and RAGE knock‐out (RAGE KO) mouse LECs (mLECs) were cultured on ECM or ECM‐AGEs for 96 h. Senescent cells in WT and RAGE KO mLECs were detected by SA‐β‐gal‐staining and representative images of WT mLECs cultured on ECM and ECM‐AGEs, and RAGE KO mLECs cultured on ECM and ECM‐AGEs are shown in (h). p16 (i) and p21 (j) levels in cell lysates were measured by western blotting (k). Data are mean ± SD of three independent experiments. ns, not significant, *p < 0.05, ***p < 0.001, ****p < 0.0001. Scale bars = 10 μm.

Next, we investigated whether blocking the interaction between AGE and RAGE could suppress LEC senescence and prevent EMT response. First, we determined the effect of the RAGE antagonist, FPSZM1, on FHL124 cell viability. When incubated with cells for 72 h, cells were unaffected at 5 and 10 μM concentrations. At a 20 μM concentration, cell viability decreased by 19%, albeit insignificantly compared to untreated cells (Figure S3). Based on these results, we used FPSZM1 at 20 μM concentration in subsequent experiments. FPSZM1 treatment to cells on unmodified ECM and ECM‐AGEs showed significant downregulation of IL‐6 release relative to their controls (*p* < 0.0001) (Figure 5a). The presence of FPSZM1 had no effect on the levels of p16 in cells cultured on unmodified ECM, whereas it significantly inhibited p16 levels in cells cultured on ECM‐AGEs (*p* < 0.001) (Figure 5b). However, unexpectedly, FPSZM1 treatment significantly upregulated p21 expression in cells cultured on both unmodified and AGE‐modified ECM. Additionally, the p21 levels were not elevated in cells grown on AGE‐modified ECM, which suggests that p21 may not play an important role in AGE‐induced LEC senescence (Figure S4a). The p38 MAPK levels were upregulated by ECM‐AGEs, FPSZM1 treatment reduced those levels, although insignificantly (Figure S4b). Cells cultured on ECM showed a smaller proportion of SA‐β‐gal positive cells relative to cells on ECM‐AGEs. The addition of FPSZM1 showed a reduction in SA‐β‐gal positive cells for both unmodified and AGE‐modified ECM (Figure 5c). FPSZM1 treatment significantly reduced levels of α‐SMA, fibronectin, and collagen I relative to cells on ECM‐AGEs only (Figure 5d–g). Primary LECs from C57BL/6J wild‐type (WT) mice cultured on ECM and ECM‐AGEs showed similar proportions of SA‐β‐gal positive cells relative to the FHL124 cells cultured on ECM and ECM‐AGEs. However, primary mLECs from RAGE knock‐out (RAGE KO) mice cultured on ECM‐AGEs showed very few SA‐β‐gal positive cells and did not differ from RAGE KO mLECs cultured on ECM (Figure 5h). In addition, p16 and p21 protein levels were significantly lower in RAGE KO mLECs cultured on ECM‐AGEs relative to WT mLECs (Figure 5i–k). Overall, these results reiterate that AGEs promote the senescence of LECs through interaction with RAGE, leading to EMT of LECs.

## 3 DISCUSSION

This study aimed to determine whether LECs undergo senescence after cataract surgery and whether AGEs in lens capsules play a role in such senescence. The migration of LECs to the posterior capsule after cataract surgery and their residence there for an extended period might make them susceptible to capsule‐AGE‐mediated senescence. In support of this notion, our study showed that LECs that had migrated to the posterior capsule and exhibited senescence were found in 4 of 12 pseudophakic human eyes. The absence of senescent cells in the posterior capsule in other samples may be due to a shorter duration after cataract surgery or inherent characteristics of the resident LECs, which may have prevented them from undergoing senescence. This heterogeneous response is interesting and may suggest individual specific traits may trigger senescence, which requires further study. Our findings that senescent LECs can induce EMT in non‐senescent cells, combined with the presence of senescent cells in certain lens capsules, may shed light on why PCO occurs in some cataract patients but not in others.

In human capsular bags cultured for 28 days, we observed an age‐dependent increase in senescent cells, suggesting that age‐related changes in the capsule contributed to LEC senescence. Further, primary LECs from a 16‐year‐old donor showed greater SA‐β‐gal staining when cultured on an aged capsule compared to a young capsule. This indicates that the aged capsule components promote LEC senescence. Given that AGEs accumulate in human lens capsules with aging (Raghavan et al., 2016), this led us to investigate whether capsule AGEs cause LEC senescence. We examined the effects of capsule AGEs on LEC senescence using a simplified ECM‐AGEs system. The levels of AGEs in the AGE‐modified ECM were comparable to those found in aged human lens capsules (Raghavan et al., 2016). Our study demonstrated that ECM‐AGEs induce LEC senescence, as indicated by a significant upregulation of p16 and p21. Our observation is analogous to several previous studies that have demonstrated AGE‐mediated senescence of other cell types (Cheng et al., 2023; Halkoum et al., 2022; Shi et al., 2019). For example, in kidney proximal tubular epithelial cells, AGE‐bearing serum albumin caused cellular senescence, which was RAGE‐dependent (Liu et al., 2014), and in atrial myocytes, AGEs induced senescence by activating the p16/Rb pathway (Zheng et al., 2022).

Another potential component in AGE‐RAGE‐mediated cellular senescence is oxidative stress. AGE‐RAGE interaction promotes ROS formation through the activation of NOX4 (Chen et al., 2018), and ROS thus formed can induce cellular senescence (Mylonas & O'Loghlen, 2022). In support of this possibility, we observed elevated levels of ROS in LECs cultured on ECM‐AGEs and a positive correlation between the ROS levels and AGE content in ECM‐AGEs. Moreover, we found that upon treatment with a NOX4 inhibitor, LECs showed significant downregulation of senescent markers. Together, these observations imply that ROS mediates AGE‐induced senescence of LECs. Whether AGEs engage RAGE in LECs, contributing to ROS production and consequent senescence, requires investigation.

Previous studies have shown that senescent cells can induce EMT‐like transition in fibroblasts (Laberge et al., 2012) and that the elimination of senescent cells inhibits the EMT in retinal pigment epithelial cells (Gao et al., 2022). In addition, several studies have shown that senescent cells contribute to tissue fibrosis (Kizilay Mancini et al., 2022; Liu & Liu, 2020; Osorio et al., 2023; Xu et al., 2020). Given these observations, we first investigated the expression of TGF‐β2 and IL‐6 in SASP, as both cytokines have also been implicated in the EMT of LECs (Ma et al., 2018; Wormstone et al., 2002). In the senescent cell population, there was a significantly greater expression of TGF‐β2 at mRNA and protein levels and a greater release of IL‐6 and MMP‐2 in cells cultured on ECM‐AGEs relative to cells cultured on unmodified ECM. Building on this, we investigated whether the conditioned medium from ECM‐AGEs‐treated LECs‐induced EMT in naïve LECs. We found that conditioned medium induced upregulation of several EMT markers in naïve LECs. These results strongly suggest that senescent cells in the posterior lens capsule promote EMT in bystander LECs and contribute to PCO.

To test our hypothesis further that AGE‐RAGE interaction is involved in LEC senescence and the subsequent induction of EMT in neighboring cells, we treated the cells with FPSZM1 to block AGE‐RAGE interaction while culturing them on either ECM or ECM‐AGE. FHL124 cells cultured on ECM‐AGEs showed higher SA‐β‐gal staining than on unmodified ECM control. The detection of SA‐β‐gal staining in the ECM control can be explained by the native mouse source of the BME, which may have been exposed to glycation prior to extraction. We previously demonstrated (Raghavan et al., 2016) that the same BME used in this study showed basal levels of AGEs, which were significantly upregulated to biologically significant levels upon glycation (Raghavan et al., 2016). A similar phenomenon was observed in Figure 5c, where cells cultured on the ECM control showed some SA‐β‐gal staining, but this was more pronounced in cells cultured on ECM‐AGEs. Therefore, our findings showing some basal staining for SA‐β‐gal activity is expected and can further support our hypothesis that the presence of AGEs promotes senescence in LECs. LECs with FPSZM1 treatment showed significant downregulation of ECM‐AGEs‐mediated SA‐β‐gal staining, p16, α‐SMA, fibronectin, and collagen I expression. Additionally, primary WT mLECs showed increased SA‐β‐gal staining and significant upregulation of p16 and p21 protein levels after culturing on ECM‐AGEs. Primary mLECs from RAGE KO cultured on ECM‐AGEs showed very little upregulation of SA‐β‐gal staining and showed no significant upregulation of p16 and p21 on incubation with ECM‐AGEs, supporting our hypothesis that AGE‐RAGE interaction is involved in LEC senescence and EMT. These results align with a previous study that demonstrated AGE‐RAGE‐mediated senescence in cells (Liu et al., 2014). A partial reduction in p38 levels was shown in cells incubated on ECM‐AGEs with FPSZM1 treatment relative to cells incubated on ECM‐AGEs only (Figure S4b). However, we found that p21 levels were significantly elevated in cells treated with FPSZM1, both in unmodified and AGE‐modified ECM (*p* < 0.05) relative to their controls. p16 and p21 have been suggested to play differential roles in inducing senescence in fibroblasts (Stein et al., 1999) and, therefore, may explain why we see differences in p16 and p21 levels in response to FPSZM1 treatment.

**SCHEME 1:**
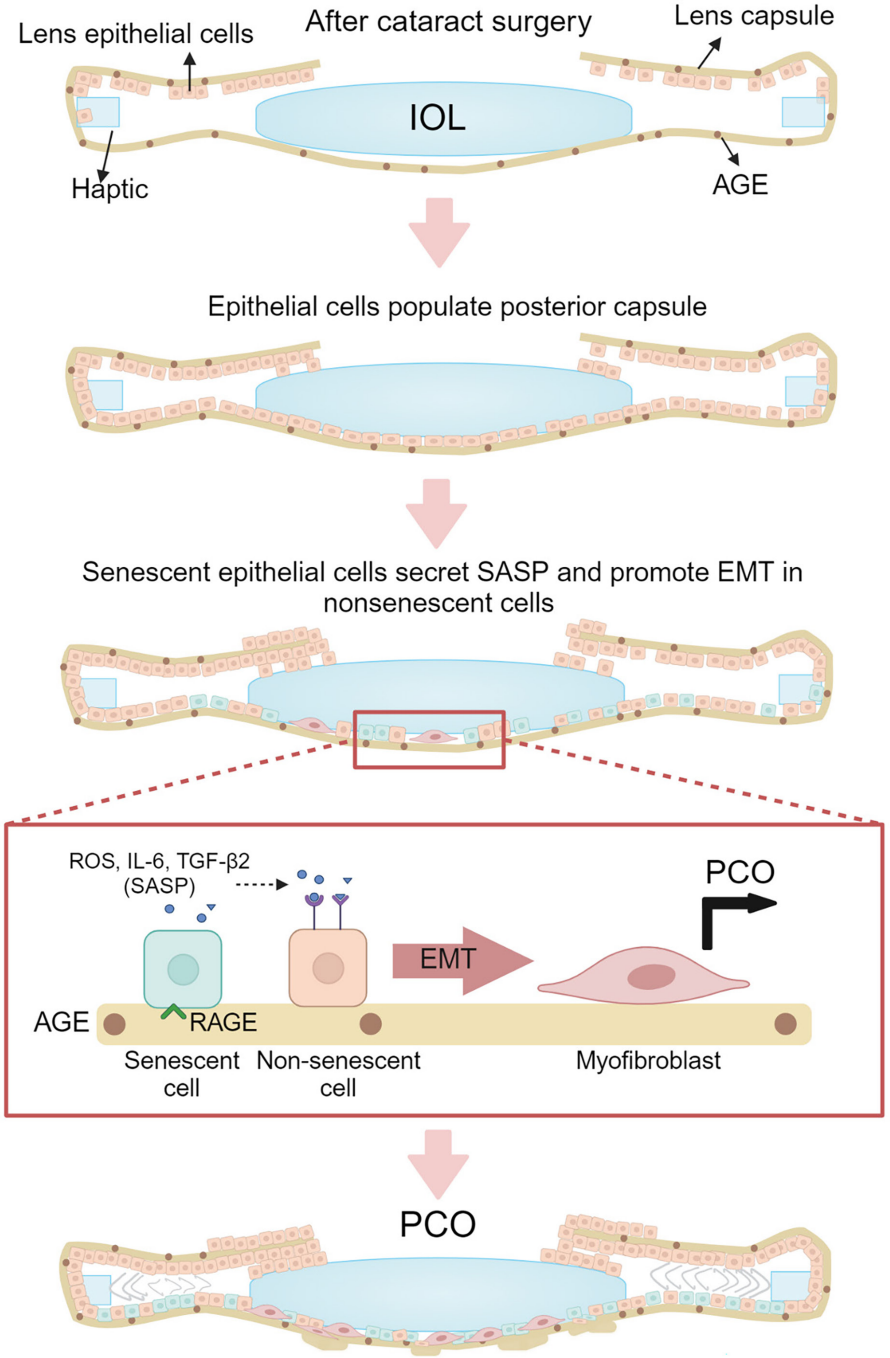
Senescent LECs promote EMT in non‐senescent LECs via a paracrine mechanism through the secretion of TGF‐β2 and IL‐6. We propose that extracellular matrix deposited from LECs that have undergone EMT and capsule wrinkling results in fibrotic PCO.

Our study showed that senescent cells are present in the posterior capsule of pseudophakic eyes, in aged human capsular bags, and in LECs cultured on aged lens capsules. In addition, we also demonstrated that ECM‐AGEs upregulate senescent markers in LECs. Based on these results, we have proposed a mechanism by which ECM‐AGEs induce senescence in LECs, resulting in SASP secretion and the promotion of EMT, contributing to PCO (Scheme 1). The discovery of senescent cells participating in the pathogenesis of many diseases and aging has prompted many investigations into the depletion of senescent cells to rejuvenate aging tissues and prevent the occurrence of diseases. Senolytic small molecules (mono or combination therapies) have shown considerable promise (Kirkland & Tchkonia, 2020; Wyles et al., 2022). Whether such senolytics could eliminate capsule‐AGE‐mediated senescent LECs and whether removing senescent cells would prevent PCO need to be investigated.

Our discoveries bear importance for fibrosis linked to aging in other tissues. Prior research has demonstrated the age‐dependent accumulation of AGEs in the basement membrane of various tissues, such as the retina and kidney (Bohlender et al., 2005; Glenn et al., 2009). Consequently, the AGE‐induced senescence in resident cells might contribute to the promotion of fibrosis and the pathogenesis of diseases in those tissues.

This work has some limitations. First, we did not investigate the role of advanced oxidation protein products (AOPPS) that could form during protein glycation. Therefore, it is possible that some of the effects we observed could be caused by AOPPS in ECM. Second, we used primary cells in our capsule bag model and the FHL124 cell line in ECM‐AGE experiments, which could limit the comparison of the effects observed between the two cell types. However, we would like to point out that the Affymetrix microarray found a 99.5% homology in the expression of 22,270 genes between FHL124 cells and the primary human LECs (Wormstone et al., 2004), providing strong support that the effects observed in the two cell types are comparable.

## 4 EXPERIMENTAL PROCEDURES

### 4.1 Preparation of human lens capsule

Human pseudophakic eyes were obtained from 45 to 92‐year‐old donors (Saving Sight, Kansas City, MO, and Lions Eye Institute for Transplant & Research, Tampa, FL). The lens capsules were isolated and pinned to a 35 mm petri dish. The IOL was removed prior to further experimentation. Cells on posterior capsules were stained for SA‐β‐galactosidase according to the manufacturer's instructions (Cat# 9860, Cell Signaling, Danvers, MA). To prepare the acellular human lens capsules, posterior capsules were isolated from young (17 to 22‐year‐old) and aged (71 to 73‐year‐old) lenses, pinned to 35 mm petri dishes, and then rinsed for 3 days with 0.85% NaCl to remove any adhering cells.

### 4.2 Human capsular bag model

Simulated cataract operations were performed to create capsular bags from human donor eyes (Liu et al., 1996; Wormstone et al., 1997) in accordance with the tenets of the Declaration of Helsinki. Approval for the study and experimental protocols (04/Q0102/57) was granted by the National Research Ethics Committee (UK). A small capsulorhexis, approximately 5 mm in diameter, was created in the anterior lens capsule, thus allowing access to the lens fiber mass, which was removed by hydroexpression. Residual lens fibers were removed by joint irrigation and aspiration. The product of this procedure is termed a capsular bag, which was then removed from the eye by dissecting the zonular fibers. The capsular bag was then secured in a 35 mm tissue culture dish using eight entomological pins (Anglian Lepidopterist Supplies, Norfolk, UK) inserted at the edge of the capsule to ensure that the capsular bag maintained its shape. All capsular bags were maintained in 1.5 mL serum‐free Eagle's minimum essential medium (EMEM) (Sigma‐Aldrich, Dorset, UK) as the standard control medium. In all cases, 50 μg/mL gentamicin (Sigma‐Aldrich) was present, and preparations were incubated at 35°C in a 5% CO_2_ atmosphere. The medium was replaced every 2–4 days, and ongoing observations of cell growth were performed using a Nikon Ti2 phase‐contrast microscope (Nikon, Tokyo, Japan) and a Nikon Qi2 digital camera (Nikon) to capture images. At the experimental endpoint (day 28), cultures were prepared and stained for SA‐β‐galactosidase according to the manufacturer's instructions (Cat# 9860, Cell Signaling, Danvers, MA).

### 4.3 Cell culture and treatment

Human primary LECs (isolated from a 16‐year‐old donor) were used from passages 3 to 5 and cultured in minimum essential medium (MEM) with 10% fetal bovine serum (FBS), 1% penicillin–streptomycin, and antimycin. FHL124 cells were cultured in MEM with 5% FBS, 1% penicillin–streptomycin, and antimycin. Cells were seeded at a concentration of 1 × 10^4^ in a 96‐well format for ROS assay and MTT assay, 5 × 10^4^ in a 24‐well format for cell attachment assay, and 2 × 10^5^ for all other experiments using a six‐well format. For culturing on ECM and ECM‐AGEs, tissue culture plates were coated in 50 μg/mL basement membrane extract (Cat #3433‐005‐0, Bio‐techne, Minneapolis, MN) in 0.1 M sodium phosphate buffer, pH 7.4 for 24 h at 37°C, 5% CO_2_. The unbound basement membrane extract was removed, and plates were then incubated with either 2 mL of 0.1 M sodium phosphate buffer, pH 7.4 or 2 mL of glycation mixture comprising 25 mM D‐glucose (Cat# G7528, Sigma‐Aldrich) 2 mM L‐ascorbate (Cat# A5960, Sigma‐Aldrich), and 250 μM methylglyoxal (Cat# 67028, Sigma‐Aldrich) (1×) in 0.1 M sodium phosphate buffer, pH 7.4 for 7 days at 37°C, 5% CO_2_. For the dose‐dependent experiment, the glycation mixture was made up to 0.25–2× concentrations. After incubation, all wells were washed thrice with ice‐cold PBS to remove unreacted glycating mix prior to cell seeding. Conditioned media from cells grown on ECM and ECM‐AGEs were collected at the experiment endpoint. One day after cell seeding on ECM or ECM‐AGEs, NOX4 inhibitor GKT136901 (Cat#534032, EMD Millipore Corp, Burlington, MA) or RAGE inhibitor FPSZM1 (Cat# 553030, Calbiochem, San Diego, CA) was freshly dissolved in DMSO and diluted in MEM (≤0.1% DMSO) and treated at a final concentration of 250 or 20 μM, respectively.

### 4.4 Primary mLECs

All animal experiments were reviewed and approved by the University of Colorado's Institutional Animal Care and Use Committee and performed in adherence to the ARVO Statement for the Use of Animals in Ophthalmic and Vision Research. C57BL/6J mice were obtained from Jackson Laboratories (Stock No. 000664, Bar Harbor, ME, USA) or bred in‐house. RAGE KO mice on a C57BL/6J background were kindly provided by Dr. A.M. Schmidt (New York University) and bred in‐house. Mice were housed with a 12:12‐h light/dark cycle and access to food and water as desired. This study included both male and female mice of ≤4 weeks of age. Isolated mLECs were cultured in MEM with 10% FBS, 1% penicillin–streptomycin, and antimycin and used from passages 2 to 4.

### 4.5 Quantitative RT‐PCR

After 48 h incubation on ECM or ECM‐AGE, RNA was extracted from cells using QIAzol lysis reagent and the RNeasy MicroKit (Cat# 74004, Qiagen, Valencia, CA). One microgram of RNA was reverse transcribed to synthesize cDNA using QuantiTect Reverse Transcription Kit (Cat# 205311, Qiagen). Quantitative PCR was performed using SsoAdvanced™ Universal SYBR® Green Supermix (Cat# 1725272, Bio‐Rad, Hercules, CA) and an iCycler iQ5 Real‐Time PCR Detection System (Bio‐Rad, Hercules, CA). The primers used were as follows: *TGFB2*: 5′‐GCCATCCCGCCCACTTTCTACA‐3′, anti‐sense: 5′‐TCCGTTGTTCAGGCACTCTGGC‐3′; *CDKN2A*: 5′‐GCTGCCCAACGCACCGAATAGT‐3′, anti‐sense: 5′‐ACTTCGTCCTCCAGAGTCGCCC‐3′; *CDKN1A*: 5′‐GACCAGCATGACAGGTGCGGAC‐3′, anti‐sense: 5′‐GGAGCATGCTGGACCAGGACCA‐3′; and *GAPDH*: 5′‐GGCTGGATGGAATGAAAGGCAC‐3′, anti‐sense: 5′‐CACAAAGGCACTCCTGGAAACC‐3′.

### 4.6 Western blot

After 96 h incubation on ECM or ECM‐AGE, cells were lysed in RIPA buffer (VWR, Radnor, PA) with a protease inhibitor cocktail (P8340, Sigma‐Aldrich), and protein levels were quantified using a BCA assay (Cat#23223/24, Thermo Fisher Scientific, Waltham, MA). Ten micrograms of proteins were separated on 12% SDS‐PAGE at 200 V for 40 min, transferred to a nitrocellulose membrane at 100 V for 80 min, and blocked using 5% nonfat dry milk (Bio‐Rad) in TBST buffer (10 mM Tris, 150 mM NaCl, and 0.1% Tween 20, pH 7.4) for 1 h. The membranes were incubated overnight at 4°C on a plate shaker with a primary antibody. The membranes were incubated with HRP‐conjugated anti‐mouse or anti‐rabbit secondary antibodies at RT for 1 h, and the signals were detected with SuperSignal Femto Kit (Pierce Chemicals, Dallas, TX). Bands were visualized using ChemiDoc™ XRS+ (Bio‐Rad, Model# Universal Hood II, Serial# 721BRO3623) and quantified using Image J, expressed relative to β‐actin control. The primary antibodies used were α‐SMA (1:2000, Cat#A5228, Sigma‐Aldrich, Saint Louis, MO). The following antibodies were from Cell Signaling Technology, Danvers, MA. p21 (1:1000, Cat#2947), p16 (1:1000, Cat#92803), MMP‐2 (1:1000, Cat#4022), fibronectin (1:500 Cat#26836), collagen I (1:1000, Cat#72026), β‐actin (1:1000, Cat#4970) and HRP‐conjugated anti‐mouse or anti‐rabbit secondary antibodies (1:2500, Cat#7076 and Cat#7074).

### 4.7 Reactive oxygen species detection

ROS production was measured at 24 h after culturing on ECM‐AGEs in the presence of NOX4 treatment. CM‐H_2_DCFDA (Cat#C6827, Invitrogen, Waltham, MA) was incubated with the cells for 30 min at a concentration of 1 μM. Fluorescence was detected by a fluorescence plate reader (SpectraMax iD3, Molecular Devices, San Jose, CA) with 484 nm excitation wavelength and 535 nm emission wavelength. ROS detection was expressed as a percentage increase over control.

### 4.8 MTT assay

Cell viability was measured by MTT assay at 96 h after culturing cells on ECM‐AGEs with and without FPSZM1. MTT (3‐(4,5‐dimethylthiazol‐2‐yl)‐2,5‐diphenyltetrazolium bromide) powder (Cat#M6494, Thermo Fisher Scientific) was dissolved in DMSO to 0.5 mg/mL, and incubated on the cells for 2 h. The MTT reagent was replaced with DMSO, and the absorbance was read at 490 nm. Cell viability was expressed as a percentage relative to control.

### 4.9 Cell attachment assay

After 24 h of culturing on ECM or ECM‐AGEs, cells were collected using 0.25% Trypsin EDTA, stained with trypan blue, and counted using TC20™ Automated Cell Counter, Bio‐Rad.

### 4.10 Flow cytometry sorting analysis

After 96 h of culturing on ECM‐AGEs, cells were labeled for SA‐β‐gal using Cellular Senescence Live Cell Activity Assay Kit (ENZ‐KIT130, ENZO, Farmingdale, NY) and sorted into a senescent and non‐senescent population using flow cytometry according to manufacturer's protocol. In brief, cells were incubated in Cell Pretreatment Solution for 2 h at 37°C then 10 μL SA‐β‐gal substrate solution was added overnight. Cells were washed in PBS thrice, collected by trypsinization, centrifuged at 128 *g* for 5 min, resuspended in 500 μL 1% FBS in PBS, and transferred to 5 mL polypropylene collection tubes. Samples were then processed by the University of Colorado Cancer Center Flow Cytometry Facility. Unstained cells were used as a negative control.

### 4.11 ELISA

The media obtained from cells cultured on ECM or ECM‐AGEs was collected at 96 h, and IL‐6 was measured using an ELISA kit according to the manufacturer's protocol (Cat#555220, BD Biosciences, Franklin Lakes, NJ). Active and total TGF‐β2 levels were measured using manual sandwich ELISA. To activate TGF‐β2, the media was treated with 2 N HCl. In brief, 25 μL of media before and after TGF‐β2 activation were incubated in a microtiter plate (Cat#3590, Corning Incorporated, Corning, NY) with 50 mM sodium carbonate (pH 9.7) overnight at 4°C. The wells were washed thrice with PBS, including 0.05% Tween‐20 (wash buffer) and blocked in 5% nonfat dry milk in wash buffer at room temperature for 2 h. The wells were washed thrice in wash buffer, and TGF‐β2 antibody (1:1000, Cat#3711S, Cell Signaling Technology) was added to the plate for 2 h. The wells were washed thrice, and HRP‐conjugated anti‐mouse antibody (1:2500) was added to the plate for 2 h. The wells were washed thrice, and 3,3′,5,5′‐tetramethylbenzidine was added to the plate for 30 min. The reaction was stopped using 2 N sulfuric acid, and the absorbance was read at 450 nm using a plate reader (SpectraMax 190, Molecular Devices, Sunnyvale, CA).

### 4.12 Statistics

All statistical analyses were performed using Graph Pad Prism 10.0.2. Data values are expressed as mean ± standard deviation (SD). Where appropriate, statistical differences between groups were calculated using unpaired *t*‐tests and one‐way ANOVA tests.

## AUTHOR CONTRIBUTIONS

RHN conceived the project. RHN, RBN, GC, M‐HN, and IMW designed the experiments; RBN, GC, M‐HN, AJOS, YMW, and IMW conducted the experiments; RHN, RBN, GC, M‐HN, AJOS, YMW, IMW, and JR performed data analysis. RHN, GC, IMW, M‐HN, and JR wrote the manuscript. RHN and IMW supervised the project.

## FUNDING INFORMATION

The work was supported by the NIH grant R01EY033915 (RHN) and an unrestricted grant from Research to Prevent Blindness, NY, to the Department of Ophthalmology, University of Colorado.

## CONFLICT OF INTEREST STATEMENT

None declared.

## CONSENT FOR PUBLICATION

All authors have agreed to the final version of this manuscript.

## Supporting information


Figures S1–S4:

## Data Availability

All data are included in the paper.
