# Supplementary material for: Lens capsule advanced glycation end products induce senescence in epithelial cells: Implications for secondary cataracts
Source: Aging Cell. 2024 Jun 21;23(10):e14249. doi: 10.1111/acel.14249 (PMC11464126; doi:10.1111/acel.14249)
Supplement: Supplementary file 1 — Figures S1–S4: [file ACEL-23-e14249-s001.docx]

**Supplementary Material**

**Lens capsule advanced glycation end products induce senescence in epithelial cells: Implications for secondary cataracts**

**Cooksley et al.,**

**
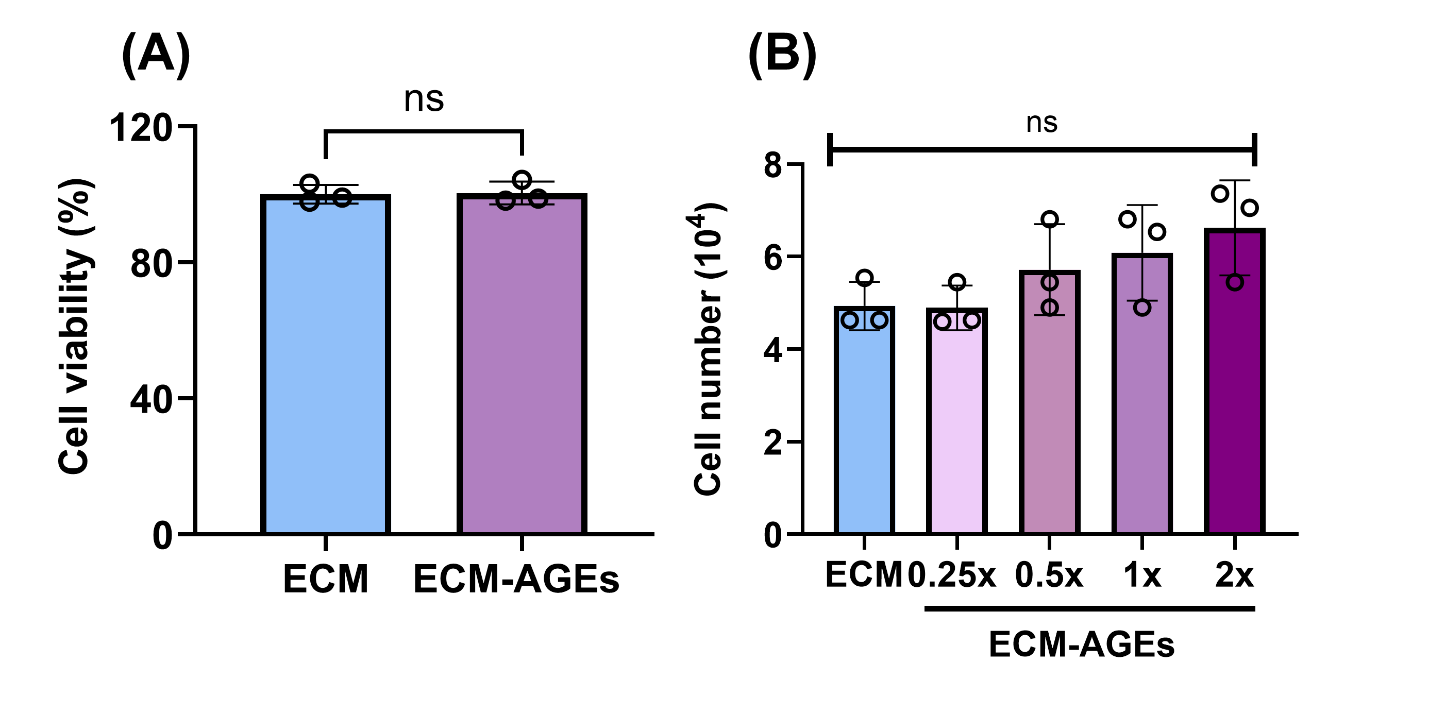
**

**Figure S1.** **Viability of FHL124 cells cultured on ECM or ECM-AGEs.** FHL124 cells were cultured on ECM-AGEs for 96 h and the cell viability was measured by an MTT assay (A). Percent reductions in viability were calculated relative to ECM control. FHL124 cells were cultured on 0.25, 0.5, 1 or 2X ECM-AGEs for 24 h, collected and stained with trypan blue and counted (B). Data are mean ± SD of three independent experiments. ns = not significant.


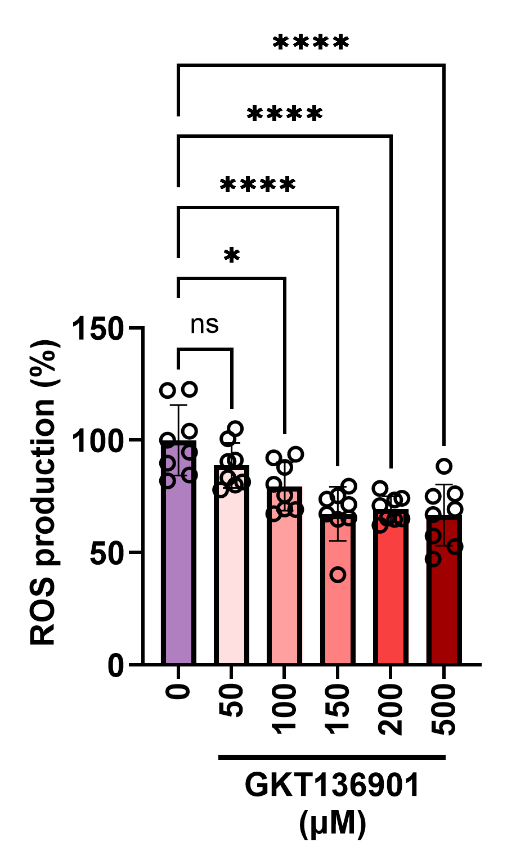


**Figure S2.** **Effect of a NOX4 inhibitor on ROS formation in FHL124 cells cultured on ECM-AGEs.** FHL124 cells were cultured on ECM-AGEs for 24 h and treated with GKT136901 (0-500 μM). ROS was measured 24 h after GKT treatment. Percent reductions in ROS were calculated relative to untreated control. Data are mean ± SD of eight independent experiments. ns = not significant. *p < 0.05, ****p < 0.0001.


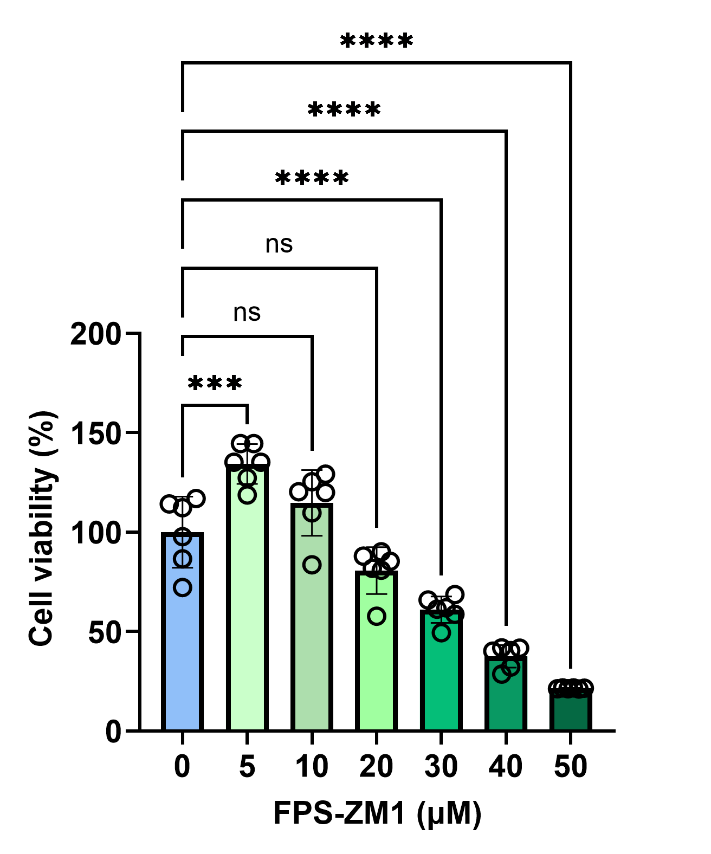


**Figure S3.** **Effect of a RAGE antagonist on FHL124 cell viability.** FHL124 cells were cultured on a 96-well plate for 24 h and treated with FPSZM1 (0-50 μM). Cell viability was measured 72 h after FPSZM1 treatment by the MTT assay. Percent reductions in viability were calculated relative to untreated control. Data are mean ± SD of six independent experiments. ns = not significant. ***p < 0.001 ****p < 0.0001.


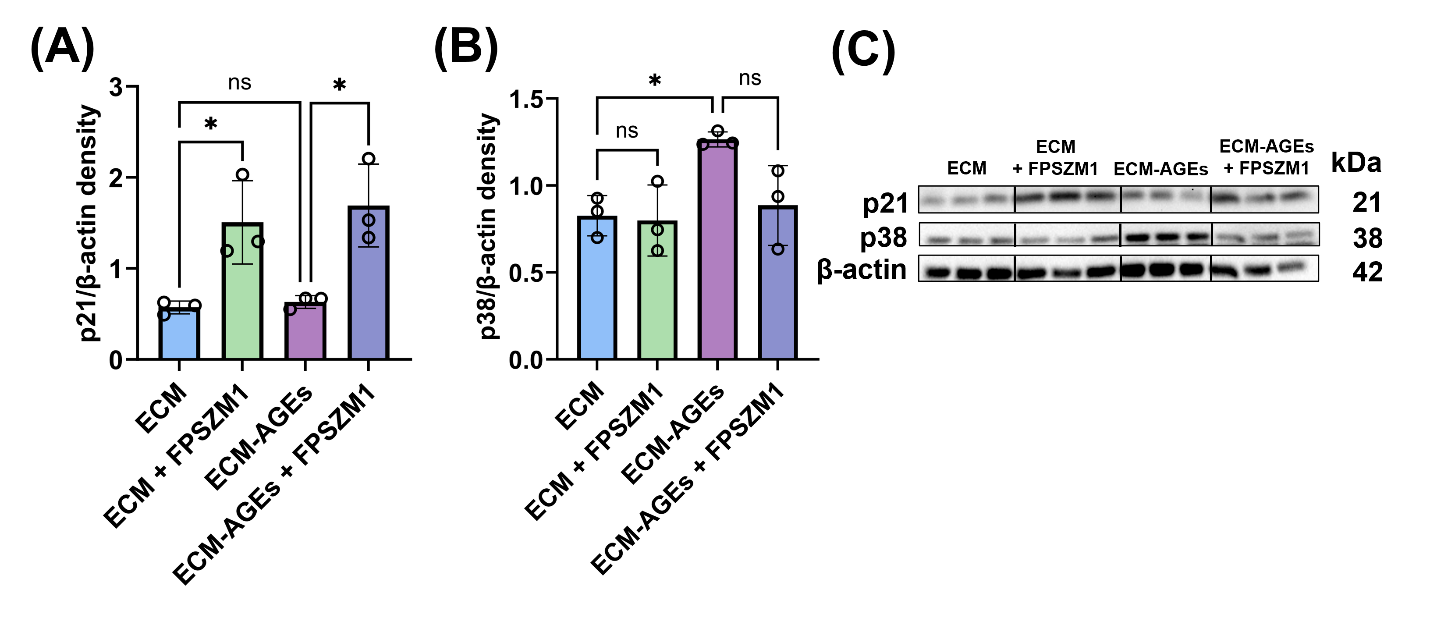


**Figure S4.** **Effect of a RAGE antagonist on senescence markers in LECs cultured on ECM-AGEs.** FHL124 cells were cultured on ECM or ECM-AGEs. After 24 h, cells were treated with or without 20 µM RAGE antagonist FPSZM1 for an additional 72 h. p21 (A) and p38 (B) were measured in cell lysates by western blotting (C). Data are mean ± SD of three independent experiments. ns = not significant, *p < 0.05.
